# Weld Defect Detection in Laser Beam Welding Using Multispectral Emission Sensor Features and Machine Learning

**DOI:** 10.3390/s25165120

**Published:** 2025-08-18

**Authors:** Amena Darwish, Manfred Persson, Stefan Ericson, Rohollah Ghasemi, Kent Salomonsson

**Affiliations:** Virtual Manufacturing Processes, School of Engineering Sciences, University of Skövde, Kaplansgatan 11, SE-541 34 Skövde, Swedenstefan.ericson@his.se (S.E.); kent.salomonsson@his.se (K.S.)

**Keywords:** laser welding, multispectral emission sensor, anomaly detection, feature extraction, feature importance, weld defect

## Abstract

**Highlights:**

- Developed a data-driven framework integrating supervised and unsupervised machine learning techniques for interpreting multispectral emission sensor data in laser welding.
- Enhanced the LSTM network with an attention mechanism to identify features related to one defect (pore—supervised learning).
- Anomaly detection in sensor signals based on features using the Isolation Forest algorithm (unsupervised learning)
- Demonstrated correlations between spectral emissions and weld defects.

**What are the main findings?**

- Data-driven framework enabling quantitative monitoring using the multispectral emission.
- Machine learning to find the correlation between spectral emissions and weld defects.

**What is the implication of the main finding?**

- Improve the in situ monitoring in weld applications.
- Explain the multispectral emission data, which make it easier to use in a decision-making system.

**Abstract:**

Laser beam welding (LBW) involves complex and rapid interactions between the laser and material, often resulting in defects such as pore formation. Emissions collected during the process offer valuable insight but are difficult to interpret directly for defect detection. In this study, we propose a data-driven framework to interpret electromagnetic emissions in LBW using both supervised and unsupervised learning. Our framework is implemented in the post-process monitoring stage and can be used as a real-time framework. The supervised approach uses labeled data corresponding to predefined defects (in this work, pore formation is an example of a defined defect). Meanwhile, the unsupervised method is used to identify anomalies without using predefined labels. Supervised and unsupervised learning aims to find reference values in the emissions data to determine the values of signals that lead to defects in welding (enabling quantitative monitoring). A total of 81 welding experiments were conducted, recording real-time emission data across 42 spectral channels. From these signals, statistical, temporal, and shape-based features were extracted, and dimensionality was reduced using Principal Component Analysis (PCA). The LSTM model achieved an average mean squared error (MSE) of 0.0029 and mean absolute error (MAE) of 0.0288 on the testing set across five folds. The Isolation Forest achieved 80% accuracy and 85.7% precision in detecting anomalous welds on a subset with validated defect labels. The proposed framework enhances the interpretability of 4D photonic data and enables both post-process analysis and potential real-time monitoring. It provides a scalable, data-driven approach to weld quality assessment for industrial applications.

## 1. Introduction

LBW is an efficient and non-contact method of joining materials used in manufacturing. In LBW, the laser beam sends a focused energy to the material in the workpiece (the specimen). Part of this energy is absorbed, and the rest of it is reflected. The absorbed energy quickly heats the material, which increases its temperature. Ultimately, the material reaches the melting point, which leads to vaporization. This vaporization generates recoil pressure, leading to a keyhole formation. A keyhole is an open hole with a cave-like shape in the molten pool, and it increases energy absorption because of the multiple reflections of the laser beam on the keyhole walls. Thus, LBW is a complex process because it is affected by various physical interactions, such as plasma and plume formation, and forces such as Marangoni flow, buoyancy, and gravity. These forces and interactions contribute to the complexity of material behavior during the welding process [1].

Due to the high complexity and stochastic behavior in the molting pool, these interactions and forces cannot be controlled; consequently, monitoring the welding process is needed to ensure weld quality. Recently, data-driven systems have been used to monitor the welding process. Researchers using these data-driven systems rely on two main approaches: vision-based monitoring [2,3,4], sensor-based monitoring, or a combination of both [5,6]. Vision-based monitoring uses images to observe the welding surface [7]. Two-dimensional images of the weld surface can help identify the locations of the laser and their correlation to the defects. Additionally, two-dimensional images are sensitive to environmental factors, such as plasma and light, which introduce noise into the image data. Furthermore, analyzing two-dimensional images has a computational cost [8].

Consequently, researchers have turned to sensor-based monitoring, such as photodiodes, as a potential solution. A photodiode collects electromagnetic feedback from the weld zone across various wavelengths. In the literature [9,10], the photodiode used divides the emission radiation into three wavelength ranges, each corresponding to one of the following: reflected laser light, thermal radiation from the weld zone, and plasma radiation, which is the temperature radiation from the plasma above the weld surface. Two studies employ a photodiode that divides the wavelength range into multiple sub-bands instead of just three [11,12]. In [11], Brüggenjürgen utilized 4D photodiodes that segment the emission radiation into 42 channels, the same sensor we used in our study. Similarly, [12] utilized a wavelength range sensor with 25 sub-bands that records wavelengths from 186 nm to 1100 nm. Although these sensors provide rich data, interpreting the high-dimensional photodiode signals remains a major challenge, as highlighted in the literature.

This study addresses that challenge by investigating whether features extracted from 4D photodiode signals can be meaningfully linked to weld quality. Our framework introduces a two-path approach: a supervised learning path, used when defect labels are available, to determine which features are most revealing of specific defects; an unsupervised learning path, used when labels are unavailable, to detect anomalous behavior by learning features of normal signals.

The novelty of this work lies in introducing a framework that uses features extracted from high-resolution 4D photodiode signals to detect weld quality issues without relying only on predefined defect labels by showing that it is possible to define threshold values that separate normal and abnormal signal data for specific weld applications. In this work, we explore whether it is possible to define signal boundaries that distinguish normal welding behavior from abnormal behavior, specifically for a given welding setup. The analysis was based on post-process sensor data, from which we identified typical signal patterns linked to known defects. These threshold values are not general-purpose; they depend on the specific process and conditions. That means, for each application, the boundaries must be defined individually. Once this is accomplished, the same values can assist during production to spot abnormal signals as they happen. This idea opens up the possibility of using photodiode sensors not only for offline analysis but also for real-time monitoring and possibly for triggering alerts during welding when a signal goes out of the expected range.

To test this framework, we conducted welding experiments on 81 samples and collected 4D photodiode data. The 4D photonics sensor spans the wavelength range from 317 nm to 1934 nm, divided into 42 channels corresponding to VIS, NIR, and laser back-reflection. We extracted statistical, temporal, and shape-based features from the raw signals to characterize welding dynamics. To analyze the relationship between the extracted features and welding parameters, we applied PCA.

This paper is structured as follows: Section 2 describes the experimental setup, including the welding system, specimen geometry, and sensor configuration. Section 3 presents the methodology. It starts with the feature extraction process and dimensionality reduction using PCA. It then describes the supervised learning model—an LSTM with attention—to identify critical time steps and feature importance associated with pore formation. Lastly, it introduces the unsupervised anomaly detection using the Isolation Forest algorithm. Section 4 provides the results of both supervised and unsupervised analyses, including insights into the spectral channels associated with weld defects and validation using longitudinal weld cuts. Section 5 concludes with key findings and highlights the potential of using 4D photonic data and machine learning for in-line monitoring of LBW. It also outlines directions for future research.

## 2. Experimental Setup

The specimen used in the experiments consisted of two overlapping AA1050 aluminum sheets arranged in a lap joint configuration. The upper sheet measured 100 mm × 25 mm with a thickness of 0.8 mm, while the lower sheet measured 100 mm × 25 mm and had a thickness of 4 mm. This geometry was selected to simulate real-world conditions for laser welding in applications involving thin-to-thick aluminum joint geometries, such as tab-to-busbar connections in battery packs [13]. All sheets were cleaned with 2-propanol before laser exposure to eliminate surface contamination.

**Figure 1:**
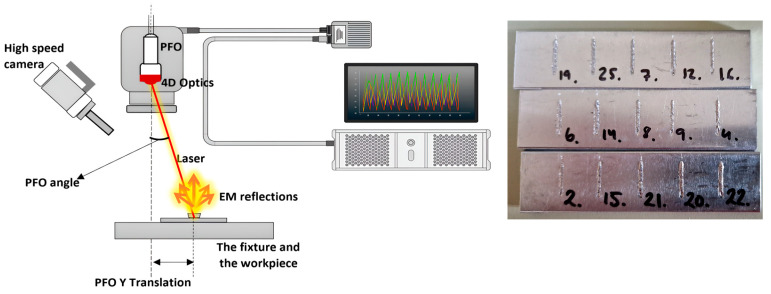
Experimental setup and an example of the specimens.

Experiments were conducted using a Laser machine (TruLaser Cell 3000, Trumpf SE & Co. KG, Ditzingen, Germany), a versatile 5-axis laser system equipped with a solid-state disk laser (TruDisk 6001, Trumpf SE & Co. KG, Ditzingen, Germany). The system included a programmable focusing optics (PFO 33-2, Trumpf SE & Co. KG, Ditzingen, Germany) laser head with a focal length of 255 mm and a collimation length of 150 mm. Figure 1 illustrates the experimental setup and provides an example of the specimen containing five weld lines. The laser emitted radiation at a wavelength of 1030 nm, delivered through a 2-in-1 fiber of a core fiber (100 µm diameter) surrounded by a ring fiber (400 µm diameter). This optical configuration resulted in an aspect ratio of approximately 1:1.7 between the fiber diameter and the focal spot size. Additionally, beam-shaping capabilities were improved by employing Trumpf’s BrightLine Weld (BLW) technology throughout the experiments.

A Box–Behnken strategy is used for the design of experiments (DoE) with four welding parameters at three different levels: laser power (3000 W, 3500 W, and 4000 W), feed rate (150 mm/s, 200 mm/s, and 250 mm/s), focus depth (−0.2 mm, 0 mm, and 0.2 mm), and inclination angle (2.74°, 4.92°, and 7.09°). The inclination angles were achieved by translating the PFO head along the *Y*-axis through translation distances of 25 mm, 45 mm, and 65 mm. Angles are calculated from Equation (1), as follows:

ϕ = tan^−1^ [(0.50 × ΔY)/292.5] × 180/π, (1)

Every combination of parameters was tested three times for 81 weld lines across 27 different experimental conditions. The literature suggested the weld parameters and correlation with pore formation and penetration defects [14,15]. Additionally, as part of a collaborative effort, a recent study by Meena demonstrated the influence of the laser beam’s angle of occurrence on pore formation. Therefore, the effect of this angle was also considered in this work [16].

### Multispectral Data Acquisition

A 4D.TWO multispectral sensor from 4D Photonics GmbH was used to capture emission data. The sensor records the spectrum in NIR and VIS ranges. It includes 16 designated channels in the VIS range and 16 in the NIR range, along with 0th-order channels for VIS, NIR, and laser back-reflection. The system supports high-precision synchronized data acquisition via a precision time protocol (PTP), resulting in a high resolution of 10 μs. The spectral wavelength ranges for the channels are categorized according to their wavelength ranges.

VIS channels: [317 + 38n] nm, where n = 0, 1, …, 15 (16 channels).

NIR channels: [1017 + 57/58n] nm, where n = 0, 1, …, 15 (16 channels).

Back-reflection laser channels: [900–1100] nm.

The wavelength is 1030 nm, which is recorded in the laser back channels and the lower ranges of the NIR spectrum. Two distinct datasets were collected with varying sampling frequencies. Dataset 0 was recorded at a sampling frequency of 5 kHz, with an integration time of 200 μs, applied to the 32 channels for both VIS and NIR wavelengths. Dataset 1 was recorded at a sampling frequency of 100 kHz, with an integration time of 10 μs, for the laser back-reflection channels and the zeroth-order channels for VIS and NIR. To capture visual features such as spatter and plume formation during the welding process, a FASTCAM NOVA S9 high-speed camera was employed. This camera recorded video at a frame rate of 6000 frames per second, with a resolution of 1024 × 1024 pixels. The high-speed imagery was utilized to verify weld defects related to spatter and visible surface anomalies on the weld.

Table 1 shows the experimental parameter values. The focus depth [mm] is the vertical distance between the laser focus and the workpiece surface. The PFO translation Y [mm] is the movement of the programmable focusing optics (PFO) along the *Y*-axis, which results in an angle for the laser welding. So, 4D photonics data were collected from the 81 weld lines. To analyze the recorded data, this study applied a feature extraction technique to allow us to identify the (statistical, temporal, and shape-based) patterns in the welding process.

## 3. Methodology

**Figure 2:**
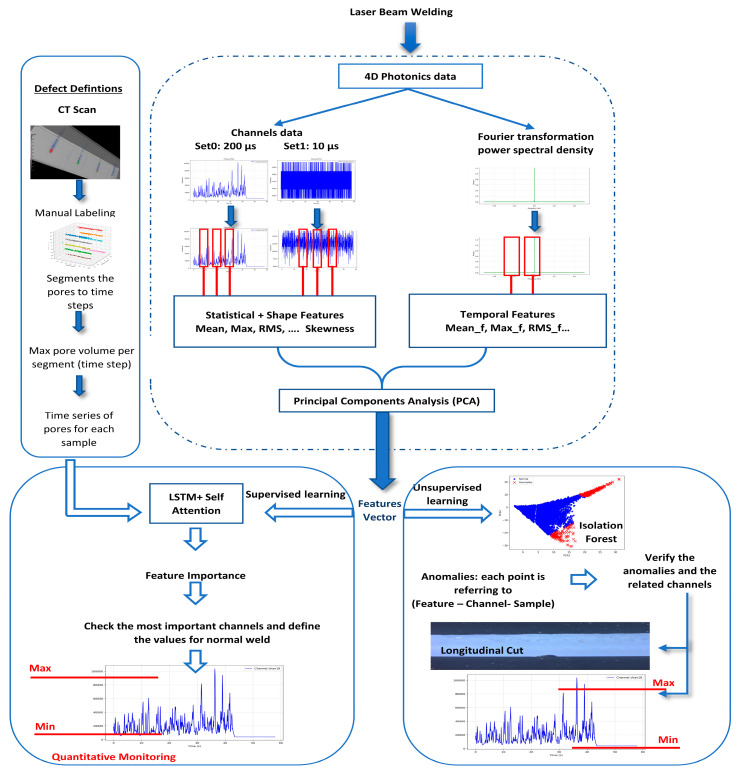
Schematic of the proposed framework for LBW monitoring and defect detection using 4D photonics data.

In this paper, we developed a data-driven approach to better understand the multispectral emission during laser beam LBW. Our goal was twofold: First, to explore how the spectral signals relate to weld defects; we chose one defect in this paper, porosity. Second, to detect unexpected anomalies from the signals. To achieve this, we combined both supervised and unsupervised machine learning methods. The supervised part is to identify specific patterns in the sensor data linked to known defects. For this, we used an LSTM model with an attention mechanism to investigate which features (from the signals) and time steps most affected pore formation. In contrast, the unsupervised part is to detect unusual behavior in the data without using labeled defects. Here, we applied the Isolation Forest algorithm. The overall framework is illustrated in Figure 2.

### 3.1. Data Processing

#### 3.1.1. Feature Extraction

Working with sensor data is vital in laser welding because the process occurs on a temporal millisecond scale, and light reflection intensities fluctuate within that same timeframe. Consequently, processing raw signal data directly and mapping electromagnetic feedback data to weld defects is challenging [10]. Researchers have verified the advantages of using extracted features from sensor data instead of raw signals [17]. They extracted both statistical and temporal features from photodiode sensor data and applied PCA to reduce the dimensionality of these features. Their study classified weld quality into one of four categories using a Support Vector Machine (SVM) classifier. This approach demonstrated that feature extraction combined with machine learning techniques can enhance weld quality.

Will et al. (2022) [17] analyzed the correlation between spatter occurrences and the extracted features to validate the integration of feature extraction into machine learning models. In their study, the FRESH library from Python was used for feature extraction. Similarly, ref. [18] used photodiode sensor data to train a Convolutional Neural Network (CNN) for classifying weld penetration levels. Their model performed real-time classification every 50 milliseconds and achieved an accuracy of 90%. More recently, ref. [19] extracted statistical and temporal features from photodiode sensor data across three channels (plasma signal, temperature signal, and back-reflection). Then, supervised learning methods (SVM and Random Forest) and unsupervised learning using an autoencoder were applied for anomaly detection. The autoencoder identified anomalies by comparing the reconstructed signals with the original ones. Furthermore, ref. [19] correlated the sensor signals with two welding defects, over-penetration and lack of connection, using feature-based analysis in defect detection.

**Figure 3:**
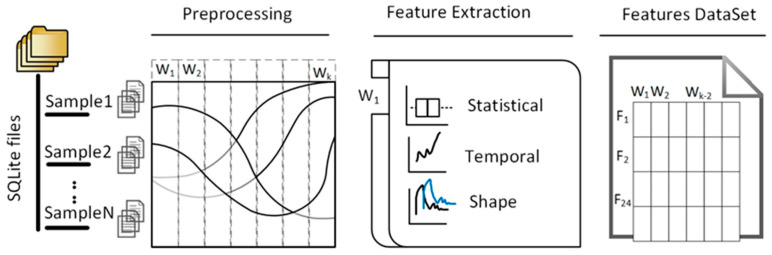
Feature extraction workflow from SQLite databases.

We extracted statistical and temporal features from each sensor channel based on these findings. These extracted features include statistical measures such as mean, variance, skewness, and frequency–domain characteristics derived from the Fast Fourier Transform (FFT), as well as shape-related metrics like crest factor and entropy. Below, we provide mathematical definitions of these key features. Based on their integration times, the data acquired from the 4D photonics system across 81-line welds were grouped into two datasets, Set_0_ and Set_1_. Each dataset comprises multiple sensor channels, which were further segmented into equal-length time windows. The defined statistical and temporal features were then calculated individually for each time window within each channel. Figure 3 illustrates the workflow of the feature extraction.

For Set_0_, Set_1_ = {S_1_, S_2_, …, S_N_}, where Si represents a sample in the dataset, and N = 81. In Set_0_, the signals have 200 μs as an integration time, while in Set_1_, the data have an integration time of 10 μs. Each sample S_i_ contains multiple channels: Si = {C_1_, C_2_, …, C_j_}, with each C_j_ representing a channel within the sample. The number of channels per sample varies, with 1 ≤ j ≤ 42.

Each channel C_j_ contains multiple windows: C_j_ = {W_1_, W_2_, …, W_k_}, where W_k_ represents different windows within the channel and k = number of windows. D(Wi) represents the signal data for window number i, where i = 0, 1, …, k.

The features were selected based on findings in the literature, where they have demonstrated strong correlations with signal fluctuations. Below, we provide mathematical definitions of these features with Equations (2)–(25).

(2) $$F_{1}W_{i}=min(D(W_{i}))$$

(3) $$F_{2}W_{i}=max\left(D\left(W_{i}\right)\right)$$

(4) $$F_{3}W_{i}=mean(D(W_{i}))$$

(5) $$F_{4}W_{i}=RMS=\sqrt{\frac{1}{k}\sum_{i=j1}^{jk}{D(W_{i})}^{2}}$$

(6) $$F_{5}W_{i}=var\;(D(W_{i}))$$

(7) $$F_{6}W_{i}=std\;(D(W_{i}))$$

(8) $$F_{7}W_{i}=power=mean\;{\left(D(W_{i}\right)}^{2})$$

(9) $$F_{8}W_{i}=peak=max\;(|D(W_{i})|)$$

(10) $$F_{9}W_{i}=peak\;to\;peak=PTP(D(W_{i}))$$

(11) $$F_{10}W_{i}=crest\;factor=peak/RMS\;(D\left(W_{i}\right))$$

Crest factor: The crest factor indicates the ratio of a waveform’s highest peak and average intensity levels.

(12) $$F_{11}W_{i}=formfactor\;=RMS/mean\;(D\left(W_{i}\right))$$

The form factor defines a periodic waveform’s shape, structure, and quality or unimodal continuous distributions.

(13) $$F_{12}W_{i}=pulseIndicator=peak/mean\;(D\left(W_{i}\right))$$

(14) $$F_{13}W_{i}=skewness=\frac{K\sum {\left(D\left(W_{i}\right)-\bar{D(W_{i})}\right)}^{3}}{(k-1)(k-2)\sigma^{3}}$$

A skewness value greater than zero indicates that the distribution has a longer tail on the right side (positive skew), while a value less than zero suggests a longer tail on the left (negative skew).

(15) $$F_{14}W_{i}=kurtosis=\frac{1}{k\;}\sum {\left(\frac{\left(D\left(wi\right)-\bar{D\left(wi\right)}\right)}{\sigma }\right)}^{4}$$

Kurtosis measures the distribution’s tailedness or peakedness. A higher kurtosis indicates more data in the tails and a sharper peak, while a lower kurtosis suggests a flatter distribution.

The second set of features consists of those extracted after applying the FFT. First, we compute the FFT of the signal and its power spectrum to obtain frequency–domain features; FFT (D(W_i_)) represents the Fourier Transform applied at the window level.

(16) $$F_{15}W_{i}=sum\_f\;(P(D(W_{i})))$$

(17) $$F_{16}W_{i}=max\_f(P(D(W_{i})))$$

(18) $$F_{17}W_{i}=mean\_f(P(D(W_{i})))$$

(19) $$F_{18}W_{i}=var\_f(P(D(W_{i})))$$

(20) $$F_{19}W_{i}=peak\_f(P(D(W_{i})))$$

(21) $$F_{20}W_{i}=skewness\_f\;(P(D(W_{i})))$$

(22) $$F_{21}W_{i}=kurtosis\_f\;(P(D(W_{i})))$$

(23) $$F_{22}W_{i}=fundamental\;frequency=first\;peak\;in\;fft\;=Peak\_f(P(D(W_{i})))$$

Equations (24) and (25) are applied directly to the signal data.

(24) $$F_{23}W_{i}=lag_{1}autocorrelation\;coefficient\;=\frac{\sum_{t=1}^{T-1}\left(x_{t}-\bar{x}\right)\left(x_{t+1\;}-\bar{x}\right)\;}{\sqrt{\sum_{t=1}^{T-1}{\left(x_{t}-\bar{x}\right)}^{2}}\sqrt{\sum_{t=1}^{T-1}{\left(x_{t+1}-\bar{x\;}\right)}^{2}}}$$

In Equation (24), T is the total time in the channel and t is the time step in the channel data. So, the autocorrelation calculates the coefficient between the time series and a one-time step-shifted series to calculate how much each time step depends on the previous one.

(25) $$F_{24}W_{i}=entropy\;(D\left(W_{i}\right))$$

Shannon [20] introduced entropy to measure the randomness within a probability distribution, which summarizes the likelihood for each point. All features are computed using Equations (2)–(25). Consequently, we obtain a collection of statistical, temporal, and shape-related features for each channel across the two datasets, Set_0_ and Set_1_.

#### 3.1.2. PCA for Dimensionality Reduction and Feature Analysis

Highly correlated and high-dimensional datasets were obtained from the previous section. Researchers in the literature, especially those who used the same sensor [11] or a very similar one [12], reduce the dimensions and deal with uncorrelated data. Similar to [17,21,22], PCA transforms the high-dimensional and correlated dataset into a new coordinate system, where the new axes, the principal components (PCs), are uncorrelated and ordered by the amount of variance they capture.

Mathematically, the PCA is represented as PCA = F.W, where F is the features and W is the loaded matrix with eigenvectors that define the directions of maximum variance. The PCA loadings matrix W is calculated by solving the eigenvalue problem for the covariance matrix.

Sum∑1/(n − 1) × F^T^ F     and          ∑W = WΛ, (26)

where W represents the eigenvectors, which represent the directions of maximum variance; Λ represents the eigenvalues, which indicate the variance explained by each PC. Each PC is a linear combination of the original features, defined by the eigenvectors in W. The weights related to Equation (27) are described in Table 2.

PCA_i_ = w_i1_ F_1_ + w_i2_ F_2_ + … + w_in_ F_n_, (27)

PCA was applied to project the high-dimensional features dataset extracted into a lower-dimensional dataset while keeping the most significant variance. We selected four principal components (PCA_1_–PCA_4_), each capturing a subset of the total variance.

Table 2 shows the PCA loading matrix. From Table 2, we can notice that PCA_1_ captures the statistical characteristics of the channels, including mean, variance, RMS, power, and peak values. These features describe the overall distribution of the channel over time. Also from Table 2, PCA_2_ is associated with the shape and distribution of the channel rather than its statistical weights. It has a high skewness, kurtosis, and entropy load, which measure irregularity, peak distribution, and randomness. PCA_3_ and PCA_4_ capture additional variations with lower weights.

After extracting the features, we have a dataset with high dimensions. Therefore, we employ PCA to reduce the number of dimensions. Now, we have the extracted features, collected from different experiments with varying welding parameters, as input. This raises several questions: Do the welding parameters influence the channel signals and affect the extracted features? Is the defect we aim to analyze measurable through sensor signals? Does the chosen weld defect vary across different welding parameters? For example, cracks (as a weld defect) occur at the final stage of solidification [23], at which point emission feedback has already been collected. This highlights the importance of analyzing the correlation between welding inputs/outputs and the extracted features.

#### 3.1.3. Analyzing the Influence of Weld Parameters on Sensor Data

In this study, weld parameters are the input, while weld quality represents the output of the welding process. To monitor weld quality, we aim to establish a connection between the input and output by analyzing features extracted from 4D photonics data. Consequently, verifying whether the chosen weld parameters and defects correlate with these extracted features is essential. Establishing this connection through the extracted features would not be feasible without correlation.

To better understand the relationship between weld parameters and extracted features, we analyze the variation in PCAs under different welding parameters. This approach identifies key parameters influencing the welding process, their role in pore formation, and the features extracted from the sensor data.

**Figure 4:**
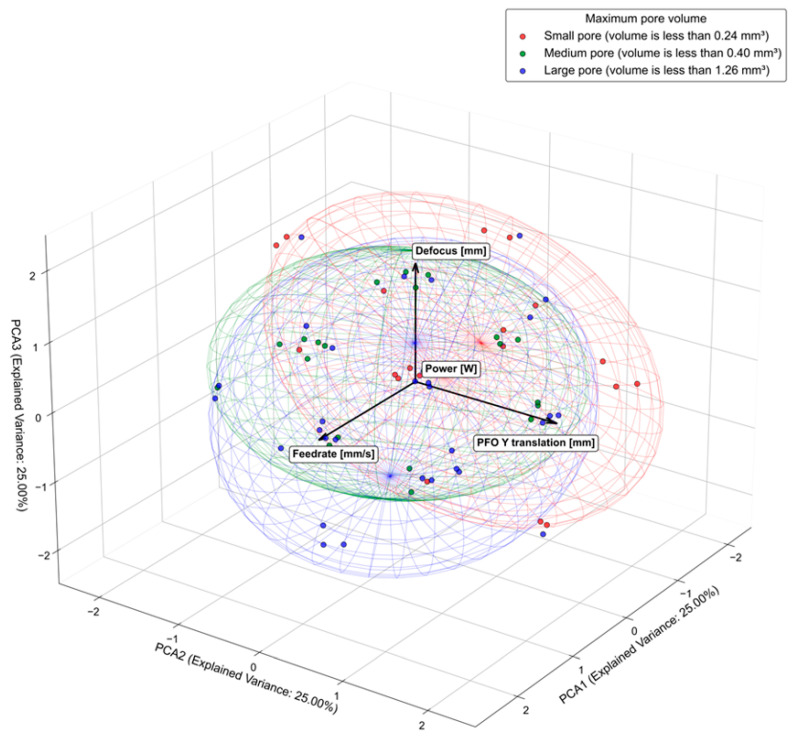
A 3D PCA biplot with confidence ellipsoids grouped by maximum pore volume. Power [W] loads on PC4 with zero loading on PC1–PC3. Therefore, its vector does not appear in the PC1–PC3 biplot. The red ellipsoid represents the small pore distribution, the green ellipsoid represents the medium pore distribution, and the blue ellipsoid represents the large pore distribution.

In this analysis, the max pore volume was measured using CT scan data, and the max pore volume for all samples was clustered into three groups: large, medium, and small. Confidence ellipsoids were computed using the following equation for the covariance matrix to visualize the distribution of each group in the PCA space:

(28) $$(\sum )=\;\;\frac{1}{n-1}\sum_{i=1}^{n}\left(x_{i}-\bar{x}\right){\left(x_{i}-\bar{x}\right)}^{\;T}$$

where x_i_ represents a data point (pore volume value) in the PCA space and $\bar{x}$ is the mean value for x_i_ for all n samples in the group. This matrix’s eigenvalues and eigenvectors define the ellipsoids’ shape and orientation. A 95% confidence ellipse was drawn. The ellipsoids represent regions where data points (pore volume) are likely to be found. A clear visualization of the distribution of different clusters (large, medium, and small pore volumes) in the PCA space is shown from one side. On the other hand, the arrows in the center of the PCA space show the correlation with the weld parameters. Longer arrows indicate a more significant contribution to the PCA’s variance, and welding parameters strongly correlate with this specific PCA component (axis) (see Figure 4).

The red ellipsoid is centered and spread equally along all axes, and we can interpret that the small pores are not affected by the process parameters. Small pores will always be present regardless of the parameters. The discussion of the pores follows in Section 3.2.1. The green ellipsoid is shifted in the PFO Y translation direction, which means that increasing the angle will accordingly increase the number of pores with a medium-sized volume (between 0.2 and 0.4). The large-pore-volume group is shifted to the bottom of the figure, so it is correlated with lower values in the defocused parameter and also lower values in the feed rate. As a result, increasing the feed rate will increase the number of pores with a large volume.

### 3.2. Modeling

#### 3.2.1. Supervised Learning

##### Data Labeling–Defect Definition (Pore Segmentation)

To guide our defect analysis, we referred to the international standards EN ISO 13913-2 and EN ISO 13919-2, which describe common weld defects in laser beam welding [24]. In this study, we focused specifically on defects related to pore formation and penetration. These standards offer useful reference examples for weld imperfections. Based on this, our goal was to examine whether the anomalies detected in the sensor data correspond to actual weld defects observed in the samples.

**Figure 5:**
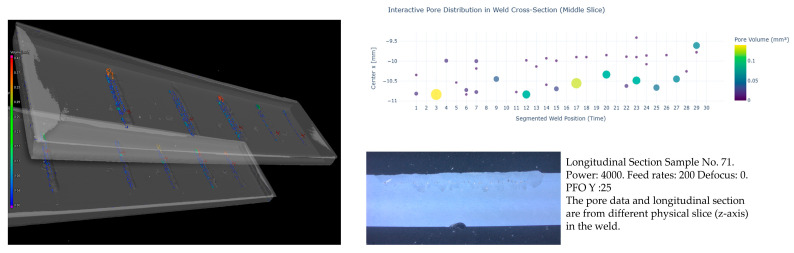
Porosity analysis: Starting with CT scans of the specimens, porosities were then analyzed using VGStudioMax. Afterwards, the data were manually labeled, projected into 2D space, and segmented into 30 time steps.

To build the labeled dataset (pore volume) over time, high-resolution volumetric data were obtained by scanning the specimens using CT. Then, the images were processed with VGStudioMax (2024.3) to extract detailed pore information, as shown in Figure 5. For porosity analysis, the identified pores were manually labeled and then segmented into 30 segments along the *Y*-axis for each specimen. This segmentation was conducted to be aligned with the 30 windows used in the feature extraction phase to ensure that each time segment meets one window in the feature dataset. As explained in Section 3.1.1, features were extracted from each window. After labeling, we had two datasets: one containing the extracted features for each channel across the 30 windows and another containing the segmented pore data for each specimen over the 30 time segments. The data within each segment were then aggregated to define the maximum pore volume per segment.

This segmentation aims to create a labeled dataset for one defect (pore volume). Therefore, it is important to identify which extracted features influence the selected defect and determine its critical time step.

##### Deep Learning (LSTM with Attention Mechanism) to Analyze Feature Time Step Importance in Predicting Pore Volume

**Figure 6:**
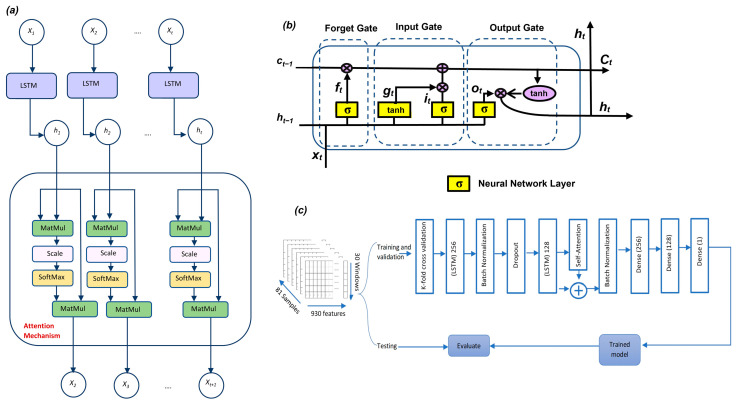
(**a**) The architecture for LSTMs and self-attention mechanism; (**b**) the architecture for a single LSTM cell with the three gates; (**c**) the deep learning architecture for the LSTM incorporating a self-attention mechanism.

LSTM networks have broad applications in general time series prediction and welding applications [25,26] because they can learn both short- and long-term dependencies in sequence data. LSTMs capture critical information in long sequences and, at the same time, avoid the gradient problem. They achieve this by using a structure in each LSTM cell, which contains three gates controlling the flow of information: the forget gate, the input gate, and the output gate. The three gates control what information should be kept or dropped as new information passes through the cell. Figure 6b shows the architecture of the LSTM cell and the three gates.

The forget gate removes unnecessary information from the last time step. It uses the current time step input and the previous time step output. The forget gate activation function (sigmoid σ) returns zero if information of the previous time step is not needed for the current time step; otherwise, it returns one (Equation (29)). The input gate determines what part of the new information in the current time step should be kept by using the sigmoid activation function. Subsequently, the cell state is made using the tanh activation function, which gives a value in the range [−1, 1] (Equations (30)–(32)). The output gate determines which part of the cell state must be passed as output to the next time step, using a σ function once more. The cell state is updated with the tanh activation function, and the final production is calculated from (Equation (33)).

(29) $$f_{t}=\sigma (w_{f}\cdot \left[h_{t-1},\;x_{t}\right]+b_{f})$$

(30) $$i_{t}=\sigma (w_{i}\cdot \left[h_{t-1},\;x_{t}\right]+b_{i})$$

(31) $${\hat{C}}_{t}=tanh(w_{c}\cdot \left[h_{t-1},\;x_{t}\right]+b_{c})$$

(32) $$C_{t}=f_{t}\;⊙\;C_{t-1\;}+i_{t\;}\;⊙\;{\hat{C}}_{t}$$

(33) $$o_{t}=\sigma (w_{o}\cdot \left[h_{t-1},\;x_{t}\right]+b_{o})$$

where w represents the weights in the gate, b is the bias in the gate, h_t−1_ is the output from the previous time step, and x_t_ is the information for the current time step; C_t_ is the cell state, and ⊙ means element-wise matrix multiplication; σ represents the sigmoid activation function.

Combined LSTMs with attention mechanisms have become increasingly popular in recent research, showing promising results in time series analysis, particularly in laser welding [27,28,29,30]. The self-attention mechanism, shown in Figure 6a, works as follows: multiply the LSTM output by itself; then, a SoftMax function is applied to correct the weights that are assigned to each time step to ensure they are not all equal. This process helps the model focus more on the most relevant time steps by giving them higher weights. Finally, the attention layer produces a square matrix, where its width and height match the number of time steps.

The output of the LSTM layer is then passed to an attention layer [27,28,29,31]. The attention layer computes alignment scores between the LSTM outputs to identify which time steps contain the most essential information related to the target output (max volume pore per segment). LSTM learns the dependencies for time steps and features to predict the pore volume per segment. By applying SoftMax (in the attention layer) to the output from the LSTM, the attention mechanism analyzes the correlation for each time step and feature to the predicted output.

The deep learning model investigates the importance of features extracted from 4D sensor signals regarding pore volume formation. The model’s prediction, generated by LSTM layers combined with an attention mechanism, is used to evaluate the model’s accuracy and validate the alignment of features with pore formation.

The dataset comprises 930 features collected over 30 windows for each of the 81 weld lines. To ensure the model’s generalizability, the dataset is divided into training and validation sets, and then K-fold cross-validation is used. Our model first includes an LSTM layer with 256 neurons to capture temporal dependencies, followed by batch normalization and dropout to prevent overfitting. The data then pass through a second LSTM layer containing 128 neurons. After the second LSTM layer, a self-attention layer with 64 neurons is added to help the model concentrate on the most important time-dependent information. As shown in Figure 6, the self-attention mechanism operates by computing alignment scores through matrix multiplication with the LSTM output. These scores are then processed through a SoftMax function, which assigns different weights to each time step, giving more importance to the most significant one, as shown in Figure 6c.

The output of the attention layer is added to the output of the LSTM layer. This integration enhances gradient flow during training and contributes to overall performance, as time steps are not treated equally after the attention is applied (more critical ones will have higher weights). Following this, a batch normalization layer standardizes the combined output. The data then flows through two fully connected dense layers, with 256 and 128 neurons, respectively, preparing it for the final prediction stage.

Finally, a dense output layer with a single neuron and a linear activation function generates the prediction (the pore volume per segment; as we mentioned, the prediction is used for evaluating the model and measuring feature and time importance). The model optimization employs the Adam optimizer, with Mean Squared Error (MSE) as the loss function. After training, the model’s predictive performance is validated using a test dataset.

#### 3.2.2. Unsupervised Learning

Since labeling welding defects is time-consuming and impractical for real-time monitoring, we employ unsupervised anomaly detection methods to identify deviations from normal welding behavior. Conducting experiments on hundreds of defective and hundreds of normal samples is critical to training a deep learning model. Therefore, an unsupervised learning method is needed to detect weld defects based on the sensor data.

The detection of anomalies mainly depends on deviations from the normal data, starting from the extracted features, assuming that most data are considered normal. Each feature from every channel and each window is regarded as a point in the data. An anomaly point is a single data point that stands out from other points. Since our data consist of these feature points and are high-dimensional, we avoid methods with high computational costs, such as those that measure the distance or density between data points.

Therefore, this study uses a partitioning-based isolation method, motivated by how the technique works. We do not need to provide the process with any prior knowledge about what constitutes an anomaly specification. The literature also supports the use of Isolation Forests for their advantages [32,33,34,35].

The Isolation Forest algorithm starts by randomly selecting a point from the data (a point in our data is a feature in a window in a channel for a one-line weld). Then, the algorithm calculates the minimum and maximum values for this point in the sub-data that the point belongs to (we can call it the point range). After that, the algorithm chooses a random split value from the point range. Then, the data are divided into two portions. This algorithm is repeated for each point until it reaches a leaf with one point or the tree reaches its maximum depth (which is a parameter we can set prior).

**Figure 7:**
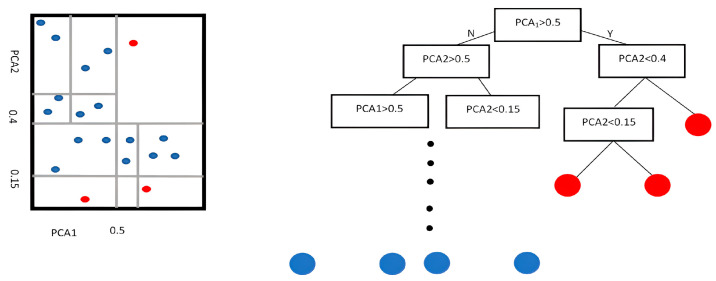
An illustrative example of the Isolation Forest algorithm: red points indicate outliers, blue points indicate normal data.

In the Isolation Forest, anomalies are efficiently isolated from the dataset, requiring fewer splits. The number of splits needed to isolate a feature is the anomaly score. The Isolation Forest algorithm defines a threshold representing the dataset’s percentage of anomalies, ensuring a thorough anomaly detection process. Figure 7 provides a clear illustration of the concept of the Isolation Forest.

This paper utilizes the Isolation Forest from the sklearn.ensemble to implement the partitioning-based isolation library in Python. After detecting anomalies, PCA is applied to reduce dimensionality and visualize high-dimensional features in a 2D plot. The segments in the tree represent decision boundaries created by the partitioning process. The Isolation Forest tree does not measure the distance between points, so the proximity of two points does not influence the anomaly results but rather their variation from the data.

## 4. Results

**Figure 8:**
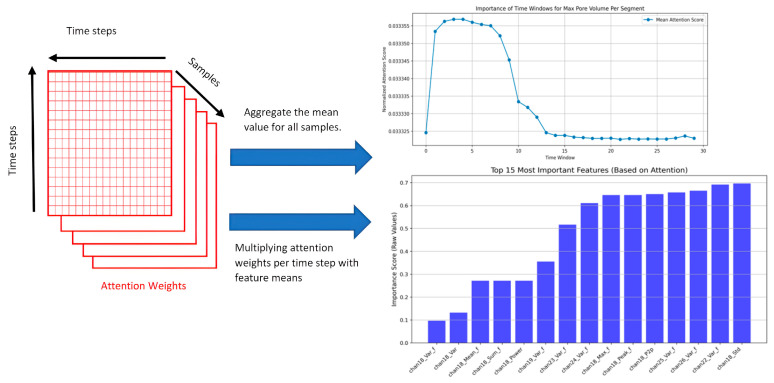
Time importance for detecting the maximum pore volume in the time segment and feature importance for detecting the maximum pore volume in the time segment.

Results from the supervised method show the importance of the feature for the chosen defect (pore volume). As illustrated in Figure 8, the features most strongly correlated with pore volume come from Channel 18, like peak-to-peak values and standard deviation. This indicates that the distance between peaks in Channel 18 impacts the volume of pore formation.

However, we cannot conclude that each peak in the channel data corresponds directly to a large pore, as the feature importance shows a moderate correlation (around 5 on a scale from 1 to 10).

Also, we should consider that the channel data are in 2D, whereas the pore formation could occur in any position in the weld in 3D space. Due to this dimensional difference, precise alignment between detected peaks in the channel data and actual pore locations is challenging. Rather than identifying exact pore positions, peaks in channel data may better indicate regions with a higher likelihood of large pores forming.

Table 3 summarizes the model’s performance, reporting both MSE and Mean Absolute Error (MAE) for the training and validation sets across all folds. The final reported values represent the average MSE and MAE over the *K*-folds.

**Figure 9:**
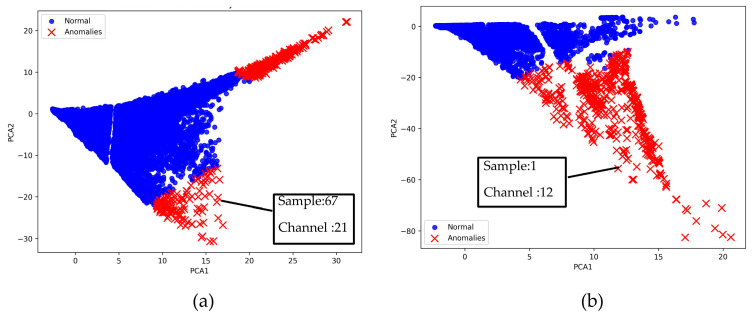
Anomaly detection using the Isolation Forest: (**a**) the anomalies in Set_0_; (**b**) the anomalies in Set_1_.

The unsupervised model (Figure 9) labeled features across all channels as either normal or anomalous. To interpret the results at the channel level, we counted how many times each channel was flagged as anomalous across all samples. This analysis revealed two distinct types of channels: those showing high variation across samples and channels that remained stable with minimal fluctuations (Table A1 in Appendix A).

Channels with frequent fluctuations may reflect noise or general variability in the welding process. In contrast, stable channels with minimal changes are more likely to capture critical information about process defects.

**Figure 10:**
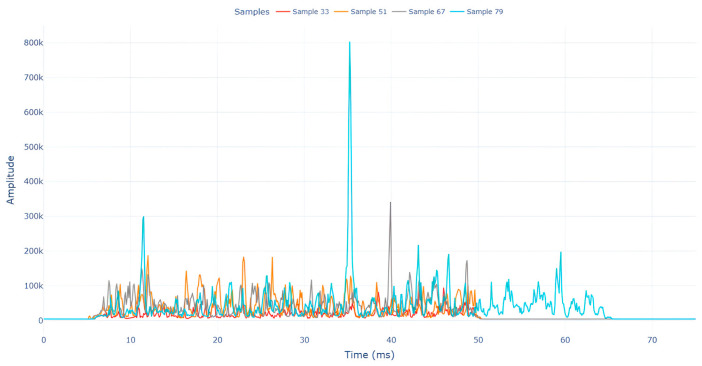
Example of Channel 18 data for four weld lines. Channel 18 is one of the VIS channels with a wavelength range of 393–431 nm.

Among the stable channels, Channel 18 shows a slight fluctuation under normal conditions. However, when changes did occur, they appeared as distinct peaks. Figure 10 shows the behavior of Channel 18 across four weld lines (Samples 33, 51, 67, and 79). Two of these samples correspond to anomaly peaks, while the other two are normal. These same samples are also listed in Table 4 and Table 5.

To determine whether these peaks reflect weld defects, a longitudinal cut was made along each weld to examine the weld depth and internal structure. This step was critical, as some samples were damaged or not cut exactly at the weld center, reducing the number of usable cases for verification.

By aligning the longitudinal cut images with the behavior of Channel 18, we can compare its variations to those of other channels that show anomalies using the anomaly detection method. Table 4 reveals that these two samples (79 and 67) have higher peaks in Channel 18, while normal samples have a maximum amplitude of about 100,000. Samples 79 and 67 display amplitudes of approximately 800,000 and 350,000 for their highest peaks, respectively. Upon reviewing the longitudinal cut images, we noted an unexpected lack of penetration that corresponds with these high peaks.

Channel 18 (394–430 nm) is associated with known aluminum and aluminum oxide emission lines. Neutral aluminum (Al I) releases at 394.4 nm and 396.2 nm, while ionized aluminum (Al II) has transitions at 407 nm and 430 nm. These wavelengths for Al I and Al II are associated with plasma formation and oxidation effects in laser welding. The occurrence of peaks in Channel 18 during lack of penetration, a common welding defect, suggests a possible link between plasma intensity, oxidation, and a lack of energy absorption, which can affect the quality of the weld.

Similarly, Channels 22 and 23 show high variations in their anomaly scores, indicating that they belong to a different category of channels characterized by significant fluctuations in the data. It is also notable that the channels in general are highly correlated, which means their extracted features are similarly correlated. This relationship is reflected in the PCA analysis shown in Table 1, where all features contribute meaningfully to the first four PCAs.

To evaluate Isolation Forest model, anomalies were aggregated at the sample level by counting how many channels were flagged as anomalous for each weld sample. Ground truth validation was conducted through visual inspection of cross-sectional images, including both longitudinal and normal cuts. However, only a limited number of samples had such images available, which restricted the extent of quantitative validation. For the samples with cross-sections, weld quality was assessed visually to determine the presence of defects. This evaluation was qualitative, as the types of defects observed varied and were not consistently labeled. Based on the subset of samples with ground truth labels, the model achieved an accuracy of 0.80 and a precision of 0.857. The ground truth annotations used in this comparison are provided in Table A2 of Appendix A.

As an unsupervised learning method, the Isolation Forest has shown promising results. Since it does not require labeled data, it can detect unexpected behavior in the weld, process instability, or significant deviations from normal data. This makes it effective for identifying material defects or unexpected environmental conditions. To maximize the benefits of this method for in situ monitoring, a set of normal weld samples can be collected to establish threshold sensor values. Once these thresholds are assigned, the system can be automated to trigger an alert and stop welding if the sensor signals exceed the threshold values.

The primary goal of using 4D sensor data is to investigate the correlation between the 42 channels, the welding process, the weld parameters, and different weld defects. Ultimately, the aim is to move toward a quantitative assessment of weld defects rather than relying on qualitative classifications that merely distinguish between acceptable (OK) and non-conforming (NOR) welds. As we seek to enable a more quantitative assessment of weld quality, by linking specific signal patterns and amplitude behaviors across selected channels to visual inspection results, we aim to better understand how defects such as pores, lack of fusion, or over-penetration manifest in the spectral data. The following tables are examples that summarize these findings so to see how certain signal behaviors correlate with observed weld conditions.

## 5. Conclusions

In this study, we developed a data-driven approach to analyze multispectral emission data captured during laser beam welding using a 4D photonic sensor. By extracting statistical and temporal features from 42 sensor channels, we explored their relationship with weld quality. Through a supervised learning model (LSTM with attention), we found moderate to strong correlations between pore volume and features extracted from Channels 18, 23, and 24. These findings suggest that certain signal patterns may indicate conditions that lead to defect formation. However, it may not be a single feature from a specific channel that plays the key role. Rather, a combination of features across multiple channels could be more indicative of pore formation. As shown in Figure 8, many features from different channels contribute similarly to pore prediction according to the LSTM with attention, pointing toward a collective signature rather than an isolated signal.

Moreover, the features most strongly correlated with pore formation tend to describe overall signal characteristics, such as standard deviation and variance, rather than signal peaks or individual values. This further highlights the challenge of linking a specific feature in a specific channel directly to pore formation. It suggests that porosity may arise from complex, distributed signal behavior rather than localized anomalies. In contrast, it is possible that other types of weld defects may show clearer relationships with specific features or channels, “a hypothesis we plan to explore in future work”.

Additionally, this study highlights the role of data-driven models, shifting the focus from experience-based knowledge to models built directly from data without prior assumptions. Instead of relying on predefined expectations, the data drives the direction of model design, leading to more adaptive and efficient anomaly detection.

Future work will explore 4D sensor data and its connection to various weld defects, including spatter and width variations. Furthermore, high-speed imaging will be incorporated to examine spatter formation and changes in weld width. This ongoing research will aid in the development of in-line monitoring systems.

## Figures and Tables

**Table 1:** Welding parameters for the welding experiments.

Parameter	
Power [W]	[3000, 3500, 4000]
Feed Rate [mm/s]	[150, 200, 250]
Focus Depth [mm]	[−0.2, 0, 0.2]
PFO Translation Y [mm]	[25, 45, 65]
Inclination Angle [deg]	[2.74, 4.92, 7.09]

**Table 2:** PCA loadings (feature contributions to each PC) according to Equation (27). This table represents the data from Set_0_.

Feature	Equation	PCA_1_	PCA_2_	PCA_3_	PCA_4_
RMS	5	0.263	0.079	0.035	−0.068
max	3	0.261	0.020	0.043	−0.122
peak	9	0.261	0.020	0.043	−0.122
std	7	0.261	0.013	−0.076	−0.058
p2p	10	0.259	0.000	−0.002	−0.112
mean	4	0.257	0.103	0.069	−0.068
sum_f	16	0.250	0.143	−0.082	0.119
mean_f	18	0.250	0.143	−0.082	0.119
power	8	0.250	0.143	−0.082	0.119
max_f	17	0.244	0.162	−0.049	0.126
peak_f	20	0.244	0.162	−0.049	0.126
var	6	0.233	0.057	−0.186	0.078
min	2	0.198	0.111	0.251	−0.139
var_f	19	0.192	0.173	−0.150	0.306
crestfactor	11	0.176	−0.272	0.169	−0.150
pulseindicator	13	0.152	**−0.334**	0.009	−0.049
autocorr	24	0.144	−0.141	**0.330**	−0.048
entropy	25	−0.133	**0.345**	0.228	0.034
formfactor	12	0.132	**−0.352**	−0.191	0.007
skew	14	0.106	−0.208	**0.430**	0.123
skew_f	21	−0.085	**0.361**	0.282	−0.019
kurtosis_f	22	−0.085	**0.359**	0.285	−0.007
kurtosis	15	0.037	−0.167	**0.416**	**0.415**
fundamental_f	23	0.013	−0.189	0.110	**0.620**

**Table 3:** Per-fold metrics (MSE and MAE) for the LSTM model.

Fold	MSE		MAE	
	Training	Testing	Training	Testing
1	0.0024	0.0045	0.0226	0.0375
2	0.0014	0.0083	0.0226	0.0461
3	0.0034	0.0003	0.0268	0.0151
4	0.0033	0.0005	0.0295	0.0178
5	0.0032	0.0010	0.0299	0.0273
Average	0.0028	0.0029	0.0263	0.0288

**Table 4:** Example of anomalous samples and their channels that appear as anomalies in the feature data.

	Sample No.: 79	Sample No.: 67
	Longitudinal cut, Sample No. 79	Longitudinal cut, Sample No. 67
	Power: 3500; Feed Rate: 150; Defocus: −0.2; PFO Y: 45	Power: 4000; Feed Rate: 200; Defocus: 0.2; PFO Y: 45
	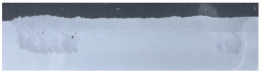	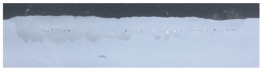
Chan18 VIS channel 393–410 nm	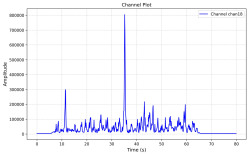	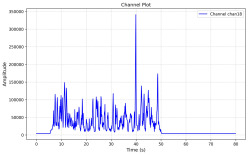
Chan23	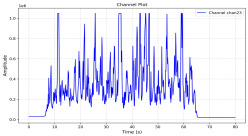	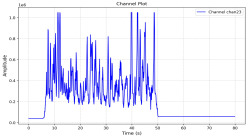
Chan24	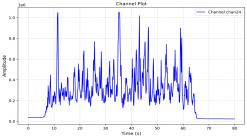	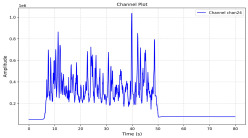
Chan63	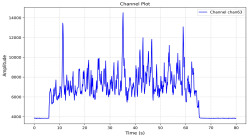	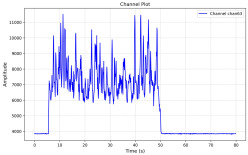
Samples 79 and 67 show unstable signals. For example, in Sample 67, Channel 24 displays sharp high-energy peaks at segment 40, with an amplitude that exceeds the values for the same channel in the normal signals. This correlates with the visible defect in the cross-section showing lack of fusion and inconsistent penetration depth. Similarly, Sample 79 shows multiple high-amplitude peaks in Channel 24, indicating instability and sudden changes in emission intensity, which aligns with the observed keyhole-shaped excessive penetration in the weld cut.		

**Table 5:** Examples of accepted weld and their channels for compression purposes.

	Sample No.: 33	Sample No.: 51
	Longitudinal cut, Sample No. 33 Power: 3000; Feed Rate: 200; Defocus: −0.2; PFO Y: 45	Longitudinal cut, Sample No. 51 Power: 4000; Feed Rate: 200; Defocus: 0.2; PFO Y: 45
	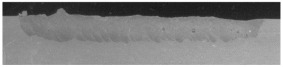	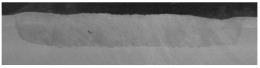
Chan18	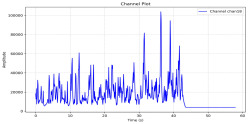	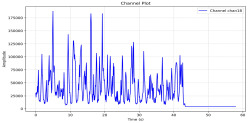
Chan23	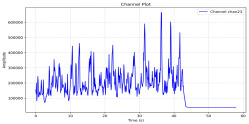	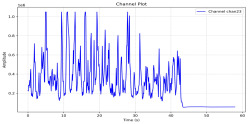
Chan24	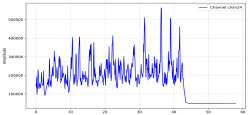	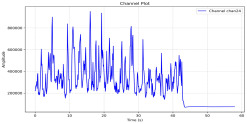
Chan63	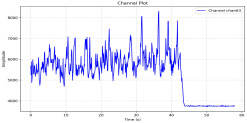	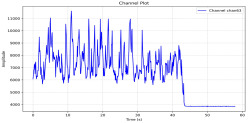
In contrast, Samples 33 and 51 demonstrate stable and smoother signal patterns, with no extreme spikes across the same channels. For example, Channel 24 in Sample 51 maintains a low-amplitude profile throughout the sequence. This consistent behavior aligns with the clean, well-formed weld cross-sections, supporting the interpretation that stable signal patterns are indicative of good welding quality. A similar observation applies to Channel 63, which shows stable signals with only minor fluctuations in these samples—especially when compared to Channel 63 in the defective samples listed in Table 4. As previously discussed, it is not only about individual spikes but about the overall behavior of the signal, which is why the extracted features are essential—they provide deeper insights into signal distribution and variability over time.		

## Data Availability

The data presented in this study are available on request from the corresponding author.

## Abbreviations

The following abbreviations are used in this manuscript:

LBW	Laser Beam Welding
VIS	Visible Spectrum (approx. 317–900 nm)
NIR	Near-Infrared Spectrum (approx. 900–1934 nm)
CT	Computed Tomography, used for pore volume analysis
PFO	Programmable Focusing Optics
ϕ	Inclination Angles
ΔY	PFO Y Translation
DoE	Design of Experiments
FFT	Fast Fourier Transform
PCA	Principal Component Analysis
LSTM	Long Short-Term Memory, a type of RNN used for time-series data
MSE	Mean Squared Error
Set_0_	Dataset collected at 5000 Hz with 200 μs integration time (VIS and NIR channels)
Set_1_	Dataset collected at 100,000 Hz with 10 μs integration time (back-reflection channels)
Wk	Window number k in a channel
D(W_i_)	Signal data in window *W_i_*
*σ*	Sigmoid activation function
⊙	Element-wise multiplication
PSD	Power Spectral Density (from FFT)
FRESH	Feature Extraction based on Scalable Hypothesis tests (Python package)
CNN	Convolutional Neural Network
SVM	Support Vector Machine
RMS	Root Mean Square
T_i_	Time step index
Eigenvector (w)	Direction of maximum variance
Eigenvalue (Λ)	Variance explained by a principal component (in PCA)

## Appendix A

**Table A1:** Shows the results of anomaly detection using the extracted features. The results are aggregated by channel name, and the number of anomalies detected in each channel is summed.

Channel	Anomaly Count in (Set_0_)	Anomaly Count in (Set_1_)
chan11	-	136
chan12	-	129
chan13	-	59
chan14	-	58
chan15	-	59
chan18	2	-
chan19	14	-
chan20	128	-
chan21	211	-
chan22	65	-
chan23	2	-
chan26	4	-
chan27	25	-
chan28	24	-
chan29	12	-
chan30	6	-
chan31	2	-
chan46	-	13
chan6	-	4
chan8	-	47

**Table A2:** Presents the results of anomaly detection based on the extracted features. The anomalies are aggregated by sample number, with the total number of detected anomalies reported for each sample.

Sample No.	Anomaly Count			Ground Truth			Model Prediction *
	(Set_0_)	(Set_1_)	Cross-Section or Longitude Cut	Defect Observed	Defect?	Weld Parameters	
23	24	3	****	The cross-section shows excessive penetration with a keyhole shape extending below the joint Keyhole length = 1571.6 μm	Yes	Power: 4000 Feed Rate: 200 Defocus: 0 PFO Y: 25	TP
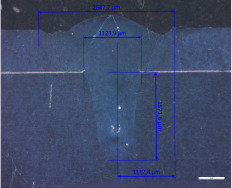
67	24	2		The penetration depth varies along the weld line, with areas of insufficient fusion between the weld metal and the base material	Yes	Power: 4000 Feed Rate: 200 Defocus: 0.2 PFO Y: 45	TP
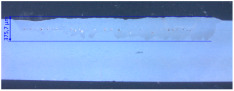
71	22	11		The penetration depth varies along the weld line, with areas of insufficient fusion between the weld metal and the base material	Yes	Power: 3500 Feed Rate: 200 Defocus: −0.2 PFO Y: 25	TP
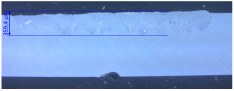
80	22	10		Small pores, but accepted weld	No	Power: 3500 Feed Rate: 150 Defocus: −0.2 PFO Y: 45	FP
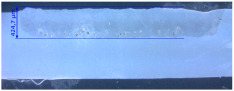
15	20	12		Undercut and lack of penetration	Yes	Power: 4000 Feed Rate: 200 Defocus: −0.2 PFO Y: 45	TP
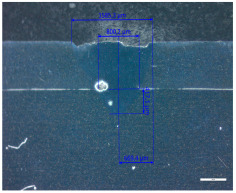
12	20	10		Pores	Yes	Power: 3500 Feed Rate: 150 Defocus: −0.2 PFO Y: 45	TP
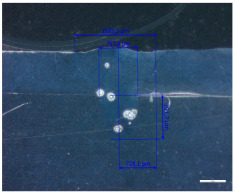
79	18	10		Lack of penetration	Yes	Power: 3500 Feed Rate: 150 Defocus: −0.2 PFO Y: 45	TP
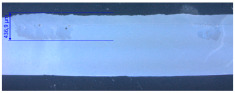
1	0	18		Accepted weld	No	Power: 4000 Feed Rate: 200 Defocus: 0 PFO Y: 65	TN
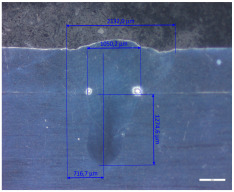
5	0	13		Accepted weld	No	Power: 3000 Feed Rate: 200 Defocus: 0.2 PFO Y: 45	TN
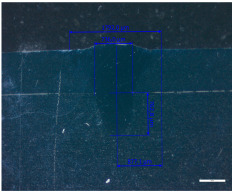
18	0	3		Accepted weld	No	Power: 3000 Feed Rate: 200 Defocus: 0 PFO Y: 65	TN
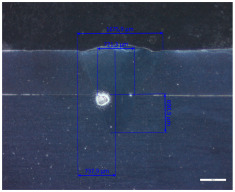
28	0	3		The penetration depth varies along the weld line, with areas of insufficient fusion between the weld metal and the base material	Yes	Power: 3500 Feed Rate: 150 Defocus: 0 PFO Y: 65	FN
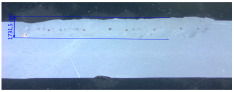
29	0	3		Accepted weld	No	Power: 3500 Feed Rate: 200 Defocus: −0.2 PFO Y: 65	TN
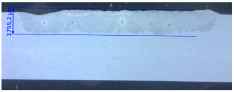
30	7	3		Accepted weld	No	Power: 4000 Feed Rate: 150 Defocus: 0 PFO Y: 45	TN
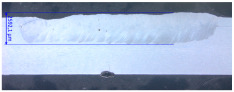
35	5	2		The penetration depth varies along the weld line, with areas of insufficient fusion between the weld metal and the base material	Yes	Power: 3500 Feed Rate: 150 Defocus: 0.2 PFO Y: 45	FN
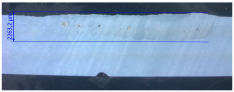
36	1	3		Accepted weld	No	Power: 3500 Feed Rate: 200 Defocus: 0.2 PFO Y: 65	TN
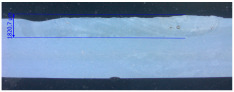
45	0	4		Accepted weld	No	Power: 3500 Feed Rate: 250 Defocus: 0 PFO Y: 25	TN
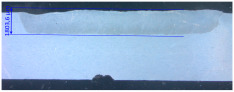
73	5	11		Accepted weld	No	Power: 3500 Feed Rate: 250 Defocus: 0 PFO Y: 25	TN
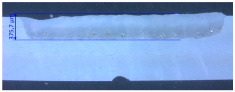
81	0	11		Accepted weld	No	Power: 3500 Feed Rate: 200 Defocus: 0 PFO Y: 45	TN
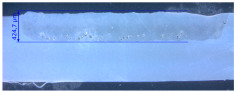
57	0	2		Lack of penetration	Yes	Power: 3500 Feed Rate: 200 Defocus: 0.2 PFO Y: 65	FN
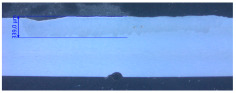
25	6	4		Accepted weld	No	Power: 4000 Feed Rate: 200 Defocus: 0.2 PFO Y: 45	TN
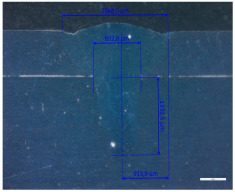

* TP: true positive. TN: true negative. FN: false negative. FP: false positive.
